# Sexual violence as a limiting factor on the perception and management of the risk of HIV in women married to migrants**^1^**

**DOI:** 10.1590/1518-8345.1141.2782

**Published:** 2016-09-01

**Authors:** Yesica Yolanda Rangel Flores

**Affiliations:** 2 PhD, Professor, Facultad de Enfermería, Universidad Autonoma de San Luis Potosí, San Luis Potosí, SLP, Mexico.

**Keywords:** Sexual Violence, Women, Migration, HIV, Aids, Risk

## Abstract

**Objective:**

to analyze the influence of sexual violence on the perception and management of the risk of HIV in women married to migrants.

**Methods:**

study with an ethnographic approach carried out in urban and rural communities. Data were obtained by methodological triangulation, with participant and non-participant observation, as well as interviews. The informants were 21 women married to international migrants. The interviews were transcribed and discourse analysis was applied to them.

**Results:**

three categories emerged from the speeches to problematize the influence of sexual violence in the perception and management of the risk of HIV: "Characterization of sexual practices in the context of migration", "Experiences of sexual violence" and "Construction of the risk of HIV-AIDS".

**Conclusion:**

women have difficulty to recognize the acts of sexual violence in their daily lives, and their perceptions of risk are not decisive in the management of the threat to which they are exposed. Therefore, it is becoming increasingly urgent that nursing problematizes the sexual violence within "steady couples", as a challenge to the promotion of healthy lifestyles.

## Introduction

Violence is a public health problem because it leads to physical, psychological and social harm; premature avoidable deaths and decline in the quality of life in little more than half the world's population^1^, and specifically, sexual violence is associated with an increased susceptibility to infection by the Human Immunodeficiency Virus (HIV) in women^2^. 

Sexual violence constitutes one of the most complex types of violence to prevent, identify and care by the nursing, in the public health sphere, as result of cultural patterns prevailing in the Latin America contexts. In such contexts, the sexual and reproductive rights of women are violated recurrently either due to the demands of conceiving the children that their husbands consider necessary or due to the fact that they are forced to accept sexual encounters under the conditions determined by their partners, even if it represents a risk to their health and lives^3^.

On the other hand, it is necessary to reflect on the speeches involving HIV in the early days of the epidemic, which linked its infection with homosexual practices and sexual services, resulting among heterosexual and monogamous women (also called "housewives") a prevailing imaginary that rejected the possibility of being infected^4^. Despite the efforts that have been made in the last decade to promote the development of awareness of these women about the risk of HIV, the historical management of this disease still remains an obstacle to the perception of its risk. 

In this context, it is worth reflecting on the situation of certain particularly vulnerable groups, such as women who are sexually involved with international migrant men and whose partnerships have been historically associated with the risk of HIV infection^5^ ^-^ ^6^. This group of women faces specific challenges in the development of perception and management of risk. On the one hand, it is recognized that their partners are particularly vulnerable to HIV during their migration processes, on the other, these women have been led to believe that they do not belong to the erroneously called "risk groups", just for being heterosexual and monogamous. 

The perception of risk determines the type of management that could be adopted^7^, and the nursing team must participate contributing so that this group of women be aware of their risk of infection, thus being able to select and manage the best strategies to confront this threat. This becomes even more complex if we consider that the perception and management of this risk do not depend solely on the access to information, but are also influenced by the conditions prevailing in social and cultural contexts. Before all else, it is necessary to assume that their experiences put them at risk, and subsequently, it is necessary to ensure that specific protection strategies are exercised with autonomy. 

In the previously mentioned context, and given the complexity of the topic addressed, it was deemed necessary to examine what is the influence of the experiences of sexual violence on the perception and management of the risk of HIV in women married to migrants.

## Methods

This was a qualitative study, with an ethnographic approach, carried out from November 2010 to November 2013, time during which two communities were visited at regular and similar periods, a rural community in the middle region and an urban community in the central area of the state of San Luis Potosi, Mexico. The locations were selected because they represent two distinct regions of the state and are characterized by a high rate of migrantion.

The relevance of conducting this study using ethnography as a research method^8^ is based on previous studies that have used ethnography to successfully address issues related to sexual practices, since the unequal power relations between genders observed in sexual practice are also evident in the daily life of men and women^9^ ^-^ ^10^. 

Participants were 21 women, and since there was no standard register of active subjects in migration, local healthcare professionals were employed as key informants or initial recruiters and later, the method of "*snowball*" was used as sampling strategy, which proved to be successfull due to the existence of networks between women married to migrants in the investigated communities. The inclusion criteria were limited to women partners of male migrants. 

It was used the application of in-depth interviews with focus on the "*history of everyday life*" and semi-structured interviews were used for the recovery of the "*narrative of risk*". The interviews focused on the history of everyday life have deepened in the activities that women and other actors with whom they socialize perform daily to subsequently, identify and analyze the experiences of violence that occur and affect their sexual and reproductive health, events that in most cases have not been identified as acts of violence.

The interpretation of the histories of everyday life allows to recover, through the narrative of these women, certain practices that due to their routinary and repetitive nature, become invisible or customary before their very eyes; attitudes, behaviors and practices that are based on subjectivities that have their origins in the imaginary and in the socially shared collective representations^11^. 

In turn, narratives of risk are a methodological proposal focused on knowing and understanding the sociocultural processes involved in the assessment of the possibility of affecting the individuals, causing them negative consequences. This method makes it possible to explain the associations that people make between people and threats, by using a series of representations that guide the interpretation of such associations^12^. 

Both the histories of everyday life and the narratives of risk were personally recorded and transcribed, and discourse analysis was subsequently applied to them in order to find recurring patterns, common themes, similarities and differences that allowed identifying the meaning units, emerging categories and qualitative domains, which were used in the theoretical analysis of collected data. 

The ethical principles of the Declaration of Helsinki and the Mexican General Health Law (MGHL) were considered for this study. This study is classified as "minimal risk" according to the MGHL since it addresses experiences presumably painful for these women, reason why it has been established from the beginning the feasibility of suspending the interviews when they affected the informants' emotional stability or when women did not show any interest to continue participating in the study. In order to prioritize the mental health of informants, a partnership was set up with psychology professionals of the State Health Services, in addition to the fact that the researcher had training in emotional support. The written informed consent was requested and total confidentiality regarding the identity of the participants was ensured. 

The project was revised, approved and supervised by the Committee on Ethics and Research of the Ministry of Health in the State of San Luis Potosí, Mexico, where the project was registered in October 2011, under protocol number 008603. 

## Results

All the women in both locations reported having experienced violence both within their marital relationship and in their wider family circle. However, difficulty has been observed in recognizing the patterns of sexual violence that they experienced within their marital life due to the "*normalization*" and "*legitimization*" that prevail in these contexts for continuous violation of sexual and reproductive rights. 

Given the wealth and abundance of information recovered from the narratives, three qualitative categories have been recovered for analysis, in order to problematize sexual violence regarding the vulnerability to HIV: "Characterization of sexual practices in the context of migration", "Experiences of sexual violence" and "Construction of the risk of HIV-AIDS". 

### Characterization of sexual practices in the context of migration 

Women have pointed out reconfigurations in sexual practices since they have reported that their partners return from the United States with greater sexual desire, fetishes and positions that they had not previously suggested, among some of the most cited innovations. These recurrent requests for sexual practices that before migration had not been mentioned get to trigger confrontations within couples, not exactly because women identify them as enhancers of their vulnerability to HIV, but because they affect their moral aspects regarding the sexual practice. 

*When he came back from there, he introduced new things in the relationship ... for example, I had never done it before ... How can I say? ... well, oral sex and I do not know how it is called when doing it from behind, so everytime he comes back, he asks me to do that, --- but I tell him that what is normal is normal and more than that I can not give him* (P.7, urban community).

However, not all women are included in the new practices of their partners, some of them report that their partners become users of sexual services after returning from the US, and many of them are sure that their partners get involved in casual or extramarital affairs.

*That day, he called me and told me that he had just done with a woman of "those" (sex worker) ... they do seek out women because they feel lonely* (P.3, urban community).

Another reconfiguration of sexual practice is the sudden interest of their partners to use condoms, which is well accepted by them, even when the request to use condom is based on unfounded arguments. 

*On one occasion, he came back and we used condom and he told me that he had to use it because the doctor there (USA) said he had flu and high cholesterol, but not because he could infect me with something, nothing but a preventive measure* (P. 7, rural community).

Speeches like the one mentioned above make it necessary to reflect on two central points, the first is to recognize the importance of developing a risk awareness in men as they start to get involved in new risk contexts; and the second is the lack of knowledge that women show about the usefulness and functioning of protection methods. 

### Experiences of sexual violence 

To characterize sexual violence is extremely complex since what prevails is a patriarchal model that considers to be normal the continuous violations of sexual and reproductive rights of people, especially women. In this context, it was found that some of these women do not experience their sexuality as a way of personal or joint satisfaction, but rather as a necessary strategy to meet the sexual desire of their partners. This is fundamentally due to an imaginary that gives them the impression that by satisfying their partners' sex drive they will be rewarded with the sexual exclusivity that society, State and Church continue to extol. 

*Doing "this" is like fulfill an obligation, sometimes he approaches me and I have no desire for him, but if I say, he gets upset because it is very, very important for him and sometimes I do unwillingly because I think, what if he seeks another women? No, it is much better to please him here as I can, though I do not feel anything good, but it does not matter, since many men usually say, "I have to seek outside what I do not find at home"* (P. 8, rural community).

This narrative reveals the attitude taken by these women who are treated as objects to serve the interests of others. They are abused by submitting themselves to forced sexual encounters, but such violence is made invisible in the framework of the imaginaries regarding the sexual obligations that women have in relation to their husbands. In this context, even though their sexuality is little or not satisfactory and they might be at risk, they continue to accept such situation because it is preferable to continue to consider themselves as "socially protected" by their partners. 

However, the "subtle" imposition of sexual encounters is not the only form of violence against them and their bodies since some men implement even more aggressive strategies to force them to accept this "*sexual obligation*", measures that may include several forms of physical, emotional or verbal violence. 

*I no longer enjoy being with him, but I agree to avoid fighting, the last time I told him no, he hit me very bad and dragged me to the road and I was all bruised. I denounced him and they came after him, but I withdrew the complaint for my children, then he was afraid and started to behave, and from there, I did not tell him no, just to avoid starting all over again* (P. 9, urban community).

### Construction and management of the risk of HIV-AIDS 

In these saturated contexts of sexual violence, special attention should be given to the fact that among these women, it prevails a kind of subjective immunity to the HIV risk. Most informants exonerate themselves from the risk, building and reproducing a stigma that has historically been instituted for those who are infected, individuals whose disease has even been demonized, being referred as a "*desired evil*" and as result of an "*addiction*" due to their sexual disfrute and "*dirty*" sexual practices.

*I think the risk is only for prostitutes and homosexuals, because these women have sex with one and another and they like do crazy things, and for the gays because they are stranger and do nasty things* (P. 9, rural community).

To recognize themselves as vulnerable to HIV represents a cultural and moral challenge for this group of women, since to assign themselves and to their partners a number of variables seems to "vaccinate" them symbolically against the disease. Among the "immunizing" variables regarding to their partners, it is highlighted the moral integrity that they attribute to their husbands, which prevent them from engaging in extramarital sexual encounters. 

*I do not think he can give me this disease, I do not think so, no one has told me that he can give it to me, we have never talked about it, but I think he does not have it, neither do I, always stuck here at home, woman is more relaxed about these things (laughs)* (P.6, comunidade rural)*.*

With regard to the management of risk, it was observed that very few women have tried to implement strategies for reducing the probability of being infected, which is evidenced in Table 1. 

**Table 1:** Acceptance and condom use by women in the communities studied in San Luis Potosi, S.L.P. Mexico, 2010-2013

		Urban women		Rural women	
		N	%	N	%
Condom use	Used	7	70	4	40
	Intended to use	3	30	0	0
	Did not use	0	0	6	60
	Did not accept to use	0	0	1	10
Reason for use	Contraceptive method	2	20	3	30
	Prevention of Sexually Transmitted Infections	5	50	1	10

Source: Direct, interview

In Table 1, it is observed that the difference regarding information on the condom is also reflected in the experiences reported about its use, a subject that shows significant differences between the two groups, prevailing a wider acceptance of its use among women from urban communities.

The factors that discourage condom use include moral aspects negatively associated with its use and the violent denial of its use by men. For some of the informants, it prevails the moral aspects negatively associated with the condom use and that prevent the assertive negotiation of these women with their partners on its use.

*I have never asked him to use condom because none of us like to use it, I think he would be angry if I asked him, I say this because I know that he would get angry at me or would even insult me (laughs*) .... ... *if he asked me to do that with condom I would say no, because I do not like it, that's for the crazy ones* (P. 20, rural community)*.*

Moreover, it was also identified that in some cases, these women were convinced of the importance of the condom use, but they had to accept the decisive denial of its use by their partners and had to live with the fear of being infected, recognizing themselves with no authority in relation to their own sexual and reproductive health. 

*He never wanted to use condom saying that it was because I am his wife, so it does not have to be like that, he does not have sex with anyone else* .... *I told him that it was because he travels from one side to another and I could not be sure, and he told me "no, it is done in the way it must be done"* (P. 9, urban community).

## Discussion

This study analyzes the influence of sexual violence on perception and management of the risk of HIV by women married to migrants. One of the most important findings of this study was the fact that no difference was observed in the experiences of violence faced by rural and urban women, which differs from the results found by other researchers^13^. However, the results of this study are consistent with those that claim that gender inequality is a reality that enhances the vulnerability of women to STIs, regardless of the features of the locations, level of education or income^14^.

The narration of a reconfiguration of sexual practices in the context of migration coincides with that reported by other studies, reinforcing the fact that be inserted in the modernist and liberal contexts seems to influence the perceptions, experiences and roles of the individuals regarding sexuality. This also seems to favor the sexual behaviors of those who are repressed in their original contexts, yet find a justification to explore new behaviors in the prevailing conditions of social, emotional and cultural pressure of the migration processes^15^

The narratives on the reconfigurations that occur in the sexual sphere show that migration can not be considered as a risky behavior, but as a dynamics that accompanies the mobility processes and enhances the vulnerability of those who migrate to STIs, as well as the vulnerability of the wives who await their return^2^.

Women have reported acts of extreme violence in their sexual practices, however these behaviors are normalized and justified based on the cultural imaginary that dictates how sexuality should be socially experienced between men and women. Such evidences reinforce the concept that for women, sexuality must be experienced more as a biological-reproductive act than an erotic and pleasurable activity. Agamben describes the asymmetry of power evidenced in the heterosexual relationships, by mentioning the categories *sacred* and *profane*, where the first corresponds to women because they are strongly associated with procreation, whereas the second is related to the attainment of pleasure and corresponds to men. In this vein, it has been considered as women's obligation to ensure the urgent sexual satisfaction of men, even if such sexual encounters cause to women great suffering^16^. 

The underestimation of the risk of contracting HIV by women is based on the trust in the sexual exclusivity of their partners and is influenced by the double standards involved in the transmission of STIs. In turn, such standards are derived from a series of cultural situations that consider the sexual practices more from a religious or mystical perspective than from the point of view of health^17^_._

In an attempt to strengthen their sense of immunity and exonerate themselves from the risk, women not only reproduce moral discourses that disqualify the different forms of sexual practice exercised in the heterosexuality, but also legitimizes a series of moral prejudgments that hinder their own perception of the risk of the disease. The support given by them to the stigma and discrimination that characterize the disease, results in a collective commitment to be achieved, since the reproduction of these imaginaries works as a disciplining mechanism that aims to convince people that there are "correct" and "incorrect" ways of relating sexually. 

Foucault argues that the sexual behavior is socially controlled by two types of devices, one of *security* and another of *disciplining*. The *safety device* (subjective immunity) works anticipating the damage, producing and supporting speeches to convince people about the importance of the practice of a heterosexual, monogamous and reproductive sexuality, to ensure the immunity against STIs in return. The *disciplining device* (stigma) is applied to publicly highlight the physical and moral damage consequences that an "*incorrect*" sexual practice brings to the social order, forcing the population to be aware of the "*tragic destiny*" of those who do not follow the regulatory standards socially established regarding sexuality^18^.

To understand the social significance that such "immunity" generates and reproduces, it is important to use the theoretical contributions of Douglas and Esposito. Douglas recognizes the existence of this "subjective immunity" as a mechanism by which individuals ignore the risks due to an excessive confidence in their environment and familiarity with their social routines. Esposito provides necessary elements to believe that this "subjective immunity" is constructed from intersubjective processes and includes the values that are a priority for each social group. In this vein, not all social groups are equal in terms of risk, housewives for example, are not "apparently" exposed to the same level of risk as homosexuals or sex workers^7^ ^,^ ^19^.

Condom use was reported as the main risk management strategy with regard to their partners, and urban women were who reported a greater awareness and habit of using such a device. This may be associated, as mentioned by other authores, with less access of rural women to formal education and with the socio-political frameworks that prevail in rural schools in Mexico, yet conservative and with teachers less able to inform about sexuality^20^

The rejection of the condom use by women may be related to the fact that their sexual practices are not experienced with satisfaction. This is based on the assumption that to enjoy a sexual practice in an erotic and pleasurable fashion involves the development of an consciousness that considers pleasure as a right and at the same time, includes a right to maintain the health and protect itself against STIs^21^.

On the other hand, this research reaffirmed the findings of other studies on moral values regarding to negotiate or accept the condom use within "steady couples", because to negotiate or accept its use may mean an authorization so that the partner accesses sexually other people^22^.

Finally, this study shows that perception of risk is not decisive in confronting the threat to which they are exposed and in risk management. Both the perception and the management depend primarily on the attitude that their partners assume in this regard, in contexts in which the patriarchal ideology discourages the acceptance of the use of protective measures and puts women at a disadvantage to decide about the conditions under which the sexual encounter takes place^23^ ^-^ ^24^. 

## Conclusion 

The women described their sexual practice as an obligation of marriage, as well as a strategy to reduce the possibility of abandonment and infidelity, or as a means to meet the biological and social function of the reproduction. As a result, they reported that they have participated in unwanted sexual encounters and under the conditions determined by their partners, in which the refusal to use a condom is frequent.

In an attempt to strengthen their sense of immunity, women often reproduce moral discourses that not only disqualify the sexual practices different of those exercised on heterosexuality, but also legitimizes a series of moral prejudices that hinder their own perception of the risk and contribute to stigmatize those who are infected.

The symbolic construction on HIV/AIDS has mainly served to stigmatize those affected by the disease, functioning as a disciplining device that punishes the individuals by the use that make of their genitals and excludes any liability of the State and society for the lack of opportunities and the inequality between men and women in the exercise of sexuality. 

The results show the importance and the need to address the risks to the health through qualitative approaches and proposals that aim to recover the experiences of individuals regarding the threats. It is also observed the need to recognize that risk does not only occur as result of a rational choice, but also as consequence of saturated contexts of cultural, social and political complexity, which make some individuals more vulnerable than others. On the other hand, it is necessary to mainstream the gender perspective in the healthcare context, recognizing the inequality of power in the sexual practices and encouraging the nursing to implement strategies aiming at promoting the empowerment of women, so that they are able to realize their risk regarding the disease and negotiate assertively the condom use with their partners.
